# Modeling the Behavior of an Underwater Acoustic Relative Positioning System Based on Complementary Set of Sequences

**DOI:** 10.3390/s111211188

**Published:** 2011-11-28

**Authors:** Joaquín Aparicio, Ana Jiménez, Fernando J. Álvarez, Jesús Ureña, Carlos De Marziani, Cristina Diego

**Affiliations:** 1 Department of Electronics, University of Alcalá, Campus Universitario s/n, 28805, Alcalá de Henares, Spain; E-Mails: joaquin.aparicio@depeca.uah.es (J.A.); ajimenez@depeca.uah.es (A.J.); urena@depeca.uah.es (J.U.); cristina.diego@depeca.uah.es (C.D.); 2 Department of Electrical Engineering, Electronics and Automatics, University of Extremadura, Facultad de Ciencias, Edificio de Física, 06006, Badajoz, Spain; 3 Department of Electronics, National University of the Patagonia San Juan Bosco/CONICET, Ciudad Universitaria Comodoro Rivadavia, Km. 4, 9005, Comodoro Rivadavia, Argentina; E-Mail: marziani@unpata.edu.ar

**Keywords:** underwater acoustic modeling, relative positioning system, complementary set of sequences, multidimensional scaling technique

## Abstract

The great variability usually found in underwater media makes modeling a challenging task, but helpful for better understanding or predicting the performance of future deployed systems. In this work, an underwater acoustic propagation model is presented. This model obtains the multipath structure by means of the ray tracing technique. Using this model, the behavior of a relative positioning system is presented. One of the main advantages of relative positioning systems is that only the distances between all the buoys are needed to obtain their positions. In order to obtain the distances, the propagation times of acoustic signals coded by Complementary Set of Sequences (CSS) are used. In this case, the arrival instants are obtained by means of correlation processes. The distances are then used to obtain the position of the buoys by means of the Multidimensional Scaling Technique (MDS). As an early example of an application using this relative positioning system, a tracking of the position of the buoys at different times is performed. With this tracking, the surface current of a particular region could be studied. The performance of the system is evaluated in terms of the distance from the real position to the estimated one.

## 1. Introduction

All systems need a good understanding of the medium in which they are deployed. Particularly, underwater medium is highly dynamic and difficult to model due to some effects as swell, turbulences or the irregular spatial distribution. Nevertheless, a propagation model is needed to test the behavior of any system prior its actual deployment. Until now, a large number of models have been proposed, based on different mathematical approaches such as ray tracing, normal mode or the parabolic equation, to name a few, where their use is suggested for different scenarios or purposes.

Ray tracing provides an intuitive approach to acoustic propagation, assuming that the energy of the wave is confined in different paths, allowing to think in rays rather than waves. This is a good assumption if the amplitude of the wave and sound speed do not change noticeably in a wavelength. Through all these years, several ray tracing codes have been developed [1–3]. These models are usually fast to compute, allow to set up the directionality of the source and can handle range-dependent sound speed profiles and bathymetry. On the other hand, they have problems with diffraction and caustics, so they are not so effective for studying bottom interactions and low frequency propagations [4].

Normal mode models are based on the integral representation of the wave equation, and provide the sound field as a sum of normal modes. They can compute transmission loss easily for a given combination of frequency, source depth, receiver depth and ranges, but they are range independent and the number of modes to compute depend on frequency, so they are recommended for frequencies below 500 *Hz* [5], unless more assumptions are considered on the environment. Two examples of these models are SUPERSNAP [4] and COUPLE, which uses coupled modes [6] to deal with range dependence.

Fast field theory is very similar to normal mode, but it uses an asymptotic expansion on the equation for the acoustic pressure field. The resulting infinite integral is evaluated by means of a fast Fourier transform and includes a branch line integral term that is usually neglected in normal modes [5]. Probably, the most famous fast field program is SAFARI [7], which can handle multiple sources and obtains the solution for all depths simultaneously, although it is not easy to use and the computation time can be very long.

Finite elements models divide the medium into a mesh. The length of the sides of this mesh is usually one tenth of the wavelength, and they intersect at nodal points. The wave equation is replaced by a system of algebraic equations, that can be solved to obtain the field at each node in the mesh. Because of that, a large computation time is required for long range and high frequency configurations [4]. There are few models based exclusively on this technique. However, it can be combined with other techniques, such as boundary integrals and wavenumber integration to solve the Helmholtz equation in a range-dependent ocean environment [8].

Other approach is the finite-difference time-domain, where a discretization of the time dependent curl equations of Maxwell is performed, and the wave propagation is simulated in the time domain. The feasibility on underwater acoustics problems was introduced in [9] using a finite-difference approximation of the Helmholtz equation, and in [10] was used for a scattering problem in an underwater medium. The Helmholtz equation can also be transformed into a boundary integral equation, and then a boundary element approximation can be used to solve it [11]. In [12], a model using the boundary element method is used to study the wave propagation in a environment with a bottom irregularity in a 3D environment.

The last main technique for modeling underwater acoustic propagation is the parabolic equation. This is an approximation of the elliptic Helmholtz equation, introduced in underwater acoustics in 1973 [4]. They can handle range dependent environments, but only narrow angles are valid, due to the approximation considered, computation time increases rapidly with signal frequency and they did not consider shear waves. Two examples of parabolic equation models are PAREQ, which dates back until 1975, and RAMS [13].

Whereas underwater modeling has been an active field since decades ago, positioning systems are a relatively new research field in underwater environments that is being very active recently. In the last decade, some systems appeared based on GPS [14,15], where this signal was used to locate some buoys on the surface. Later on they used this information to obtain the position of a submerged object using underwater acoustics, so different sensory systems were used. Other approach using GPS can be found in [16], where the GPS clock were used to synchronize different buoys and were also used as an emitted signal, to obtain the time difference of arrival, checking the clock time when the signals arrive at a buoy. More recent advances include the use of different techniques, like [17], which describes a new synchronized intelligent buoy to be used in long baseline schemes, where some beacons with known positions are used to locate some object. In [18], a particle filter were used to track a moving source using different sensors whose positions were assumed to be known.

On the other hand, applications like sonars or positioning systems need accuracy in the estimation of times of flight. To achieve this accuracy, coding the emitted signal is a good solution, obtaining these times of flight by means of correlation processes. This technique has been widely used in airborne environments [19–22], but not so much in underwater environments, being an area of research with much schemes to explore.

As the bottom interaction is not the main purpose of this work, a high frequency signal has been used (20 *kHz*), and the environment is not restricted to very short ranges, ray tracing was the optimal technique to use due to its simplicity and accuracy for high frequency signals. In this work, an underwater acoustic propagation model based on the ray tracing technique is presented. In addition to the usual phenomena, such as geometrical spreading, absorption and energy loss at the surface and the bottom, the model also considers the effect of swell in the surface-reflected signals, and obtains the impulse response for a dynamic channel.

The model has been developed in Matlab. A cluster of computers has been used to perform a statistical study of the behavior of the relative positioning system. This system uses acoustic signals coded with CSS to obtain the times of flight. With these times of flight and knowing the sound speed value, the distances between the buoys are obtained and fed to the MDS technique, which obtains the relative positions of the buoys knowing only the distances between them.

This system does not need GPS measurements, nor prior information regarding the position of the buoys. Every buoy is also capable to locate itself and the others. Additionally, it would be an inexpensive solution as well as a system easy to deploy and use. As an application example, the relative positioning system is used to track the movement of surface buoys due to a surface current, although its main advantage would be in the positioning of submerged objects. The distance between the real position and the estimated position is used as a performance criteria, which allows to study the feasibility of this kind of system.

The rest of the paper is organized as follows. In Section 2 the fundamentals of the propagation model are presented. Section 3 describes the relative positioning system, the CSS coding scheme and the MDS relative positioning algorithm. Section 4 shows some simulated results for the behaviour of the system, and Section 5 outlines the conclusions and future work.

## 2. Underwater Acoustic Propagation Model

In this section, the underwater acoustic propagation model is presented. First, some fundamentals about the ray tracing technique are given. Then, the main parameters involved in acoustic propagation are described in detail.

### 2.1. Ray Tracing

Although this model is based on the ray tracing technique, it does not solve the differential equations, but rather uses a geometrical approach. As stated before, ray tracing assumes that the energy of the wave is confined in different paths or rays. This assumption is valid for high frequencies, so it is a good choice for modeling kilohertz signals and above.

The water column is assumed to be stratified, obtaining a large number of layers. In each layer, sound speed is considered to be constant, but it can change from one layer to the next one. Thus, a ray path will follow a straight line within each layer [23], but the angle of that path can change due to the variation of the sound speed in the next layer, as it is stated by Snell Law, causing the curvature of the ray. The thickness of these layers is a trade-off between the desired accuracy and the algorithm computation time.

The rays will propagate through the medium, and they will lose energy due to different processes. This model considers the energy loss caused by geometrical spreading, absorption and rebound losses at the surface and the bottom. This transmission loss is computed for each ray that arrives at the receiver, which is called an eigenray. Both the sea surface and the bottom are considered flat for computing the ray paths. Nevertheless, wind speed is included in the model, which will cause a Doppler spread in the surface-reflected signal due to the swell.

The eigenrays are obtained following an intensive search. First, a small number of rays (typically between 20 and 40 rays) are launched. Then, the number of rebounds of two adjacent rays are compared. If they have the same number of rebounds, the final positions in the water column at the receiver end of these rays are compared with the receiver depth. If the receiver is placed between the two rays, it can be assumed that there will be a ray between them that will hit the receiver. The properties of this eigenray are obtained interpolating the values of the other two. However, if the receiver is not placed between them, there will be no eigenray. Another possible situation is when two adjacent rays do not have the same number of rebounds. If that is the case, another 10 rays are launched between them, searching for the edge rays with the same properties than the other two. Then two beams will be obtained out of one, and the process above is repeated. This intensive search is performed for each two adjacent rays.

**Figure 1.**
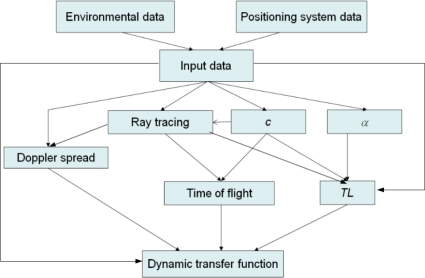
Block diagram of the propagation model.

The block diagram of the model is shown in Figure 1. The inputs are environmental parameters, such as temperature, depth, salinity, bottom and water densities, wind speed or sound speed at bottom material; and the positioning system parameters, such as signal frequency, hydrophones’ depth, the aperture angle of the transducer or the position of the buoys. With all these data, the sound speed can be computed, as well as the absorption coefficient, *α*. Knowing the sound speed profile, the model performs a ray tracing in the considered environment, and computes the Doppler spread. With the information from the ray tracing and knowing the sound speed profile, the times of flight from the different arrivals are obtained, whereas the transmission loss (TL) is computed knowing the absorption coefficient, sound speed in water and in bottom material and using information from the ray tracing, such us the distance traveled by the rays, as well as some of the inputs of the model, like wind speed or signal frequency. The dynamic transfer function is then obtained using the internal Matlab function *rayleighchan*, which need as inputs the times of arrival for the different eigenrays as well as their transmission loss, the sampling frequency and the Doppler spread.

During the development of the model, its results were compared with the ones provided by the ray tracing code Bellhop [1], obtaining similar results. Additionally, in [24] a comparison between the model results and an experiment result found in the literature was conducted, showing a good performance.

### 2.2. Sound Speed

Sound speed in water *c*, can be theoretically obtained from the linear wave equation as Equation (1) [25]:

(1) $$c^{2}=\gamma \frac{B_{T}}{\rho_{0}}$$

where γ is the adiabatic index, *B_T_* is the isothermal bulk modulus and *ρ*_0_ is the equilibrium density. However, the variations of these parameters with temperature and depth are not easy to predict, so in the last decades several empirical formulas have been given. In this propagation model, the equation by Chen and Millero has been used, Equation (2) [26], due to its wide range of valid inputs for salinity (*S*), temperature (*T*) and pressure (*P*).

(2) $$c(S,\;T,\;P)=C_{w}(T,\;P)+A(T,\;P)S+B(T,\;P)S^{3/2}+D(T,\;P)S^{2}\;\;\;\left({\mathrm{ms}}^{-1}\right)$$

with

$$\begin{array}{lll} C_{w}(T,\;P) & = & \left(C_{00}+C_{01}T+C_{02}T^{2}+C_{03}T^{3}+C_{04}T^{4}+C_{05}T^{5}\right)\\ & + & \left(C_{10}+C_{11}T+C_{12}T^{2}+C_{13}T^{3}+C_{14}T^{4}\right)P\\ & + & \left(C_{20}+C_{21}T+C_{22}T^{2}+C_{23}T^{3}+C_{24}T^{4}\right)P^{2}\\ & + & \left(C_{30}+C_{31}T+C_{32}T^{2}\right)P^{3}\\ A(T,\;P) & = & \left(A_{00}+A_{01}T+A_{02}T^{2}+A_{03}T^{3}+A_{04}T^{4}\right)\\ & + & \left(A_{10}+A_{11}T+A_{12}T^{2}+A_{13}T^{3}+A_{14}T^{4}\right)P\\ & + & \left(A_{20}+A_{21}T+A_{22}T^{2}+A_{23}T^{3}\right)P^{2}\\ & + & \left(A_{30}+A_{31}T+A_{32}T^{2}\right)P^{3}\\ B(T,\;P) & = & B_{00}+B_{01}T+\left(B_{10}+B_{11}T\right)P\\ D(T,\;P) & = & D_{00}+D_{10}P \end{array}$$

where the values for the constants *C_ij_*, *A_ij_*, *B_ij_* and *D_ij_* are given in Table 1 [26]. This equation can be used for temperatures between 0 °*C* and 40 °*C*, pressures between 0 *bar* and 1, 000 *bar* and salinities between 0‰ and 40‰, which cover all kind of scenarios in this medium.

Sound speed is computed by Equation (2) for each layer of the water column, knowing the temperature profile and salinity, whereas pressure is obtained from the Leroy–Parthiot equation [27], which provides a depth to pressure conversion. A sound speed profile is then obtained and, considering it, the model can be applied both in shallow waters and deep waters. This sound speed profile is assumed to be constant in the present environment under study.

### 2.3. Transmission Loss

There are three main contributions to energy loss of an acoustic wave in water: geometrical spreading, absorption and rebounds at the surface and the bottom. This energy loss must be computed for each ray.

Geometrical spreading *T L_geo_*, is caused by the expansion of the acoustic wave through the medium. Depending on the geometry of the channel, there are two types of spreading: spherical and cylindrical. Geometrical spreading is computed by Equation (3), where *r* is the distance traveled by the ray and *k* is a constant which value is 10 for a cylindrical spreading (typical for shallow water environments) or 20 for a spherical spreading.

(3) $$T\;L_{\mathrm{geo}}=\mathrm{klog}\;r\;\;\;(\mathrm{dB})$$

As the wave travels through the medium, some of its energy passes to the water as heat: this is the absorption loss, *T L_abs_*, Equation (4). This loss depends as well on the distance traveled by the ray *r*, and the absorption coefficient *α*.

(4) $$T\;L_{\mathrm{abs}}=\alpha r10^{-3}\;\;(\mathrm{dB})$$

There are several empirical formulas to compute the absorption coefficient. In this work the Francois–Garrison equation has been used, Equation (5) [28], due to its wide range of valid input frequencies, between 100 *Hz* and 1 *MHz*.

(5) $$\alpha =\frac{A_{1}\;P_{1}\;f_{1}\;f^{2}}{f^{2}+f_{1}{}^{2}}+\frac{A_{2}\;P_{2}\;f_{2}\;f^{2}}{f^{2}+f_{2}{}^{2}}+A_{3}\;P_{3}\;f^{2}\;\;\;\left({\mathrm{dBkm}}^{-1}\right)$$

where *A_i_* and *P_i_* are functions that can depend on pH, salinity, depth, temperature and sound speed; *f*_1_ and *f*_2_ are the relaxation frequencies of boric acid and magnesium sulfate, respectively; *f* is the wave frequency, all of them given in *kHz*.

The model can compute the bottom loss *T L_bot_*, by two means. One of them is the Rayleigh model [29], with which bottom loss is obtained by Equation (6):

(6) $$T\;L_{\mathrm{bot}}=10\log{\left[\frac{q\;\text{sin}\;\theta -{\left(n^{2}-{\text{cos}}^{2}\theta \right)}^{1/2}}{q\;\text{sin}\;\theta +{\left(n^{2}-{\text{cos}}^{2}\;\theta \right)}^{1/2}}\right]}^{2}\;\;(\mathrm{dB})$$

where *q* = *ρ_b_/ρ_w_* and *n* = *c_w_/c_b_*, *ρ_b_* is the bottom density, *ρ_w_* is the water density, *c_w_* is the sound speed in water, *c_b_* is the sound speed in bottom material and *θ* is the angle of incidence. This model is one of the simplest to compute the bottom loss, and there will be no loss for an angle of incidence lower than the critical angle. Another option included in the model to compute the bottom loss is to consider a constant value for *T L_bot_* for each rebound at the bottom, depending on the bottom material.

As for the surface rebounds, one of the simplest equations to obtain the surface loss *T L_sur_*, is the Beckmann–Spizzichino formula, in the form given by R. Coates in Equation (7) [30],

(7) $$T\;L_{\mathrm{sur}}=10\log\left(\frac{1+{\left(f/f_{1}\right)}^{2}}{1+{\left(f/f_{2}\right)}^{2}}\right)\;-\left(1+\frac{90-w}{60}\right){\left(\frac{\theta_{s}}{30}\right)}^{2}\;\;(\mathrm{dB})$$

where *f* is the signal frequency in *kHz*, *w* is the wind speed in knots, *θ_s_* is the angle of incidence at the surface in degrees, $f_{1}=\sqrt{10}f_{2}$ and *f*_2_ = 378*w*^−2^.

Adding together all the terms introduced before, the total transmission loss for each eigenray can be obtained by Equation (8), where the minus sign corrects the positive value of these magnitudes, and *n_bot_*, *n_sur_* are the total rebounds suffered by the ray at the bottom and the surface, respectively.

(8) $$\mathrm{TL}=-{\mathrm{TL}}_{\mathrm{geo}}-{\mathrm{TL}}_{\mathrm{abs}}+n_{\mathrm{bot}}{\mathrm{TL}}_{\mathrm{bot}}+n_{\mathrm{sur}}{\mathrm{TL}}_{\mathrm{sur}}$$

### 2.4. The Dynamic Effect of Swell

It has been previously stated that the surface is flat for computing the ray paths. However, the swell will cause a moving surface and a motion of the reflection point. This motion will cause a Doppler spread *B* in the surface-reflected signals, given by Equation (9) [31],

(9) $$B=0.0175\left(\frac{f}{c}\right)w^{3/2}\text{cos}\;\theta_{s}\;\;(\mathrm{Hz})$$

where *f* is the signal frequency, *c* the sound speed, *w* the wind speed in *ms*^−1^ and *θ_s_* the angle of incidence to the surface. This Doppler spread will provide a dynamic transfer function, which follows a Rayleigh distribution of amplitudes and causes a random phase shift to the surface-reflected signals.

## 3. Relative Positioning System

One of the main advantages of relative positioning systems is that only the distances between all the buoys are needed to obtain their positions. In this work, the relative positioning system consists of four buoys. Two of them are anchored at a fixed position: one of them is considered the origin of the coordinate system, so its position in the horizontal plane will always be (0, 0) *m*. The other fixed buoy helps define the axes of the Cartesian coordinate system by forming a line with the other buoy. Finally, the other two buoys can freely move through the sea surface.

**Figure 2.**
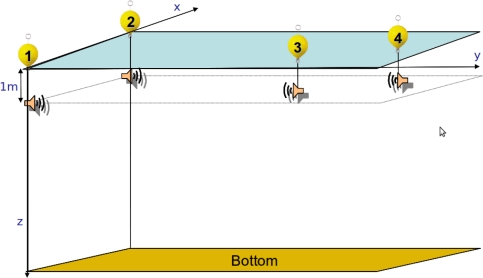
Positioning system configuration.

At 1 *m* depth in each buoy there is a hydrophone, acting both as an emitter and receiver. The signal emitted by these hydrophones is supposed to be omnidirectional in the horizontal plane, but limited between *±*20° in the vertical plane. Figure 2 shows the positioning system configuration.

For this simulated example, the buoys are supposed to be perfectly synchronized, e.g., by a RF link. As the errors in the system are in the order of a meter, as it is shown in Section 4, the system will be robust to synchronization errors up to approximately two hundred microseconds. These synchronization errors would cause a positioning error of the order of few decimeters. This tolerable synchronization error is greater than those found on the literature [32,33] for RF synchronization. An emission every three minutes is considered.

### 3.1. Coding Scheme

In order to obtain the times of flight more accurately, the acoustic signals are coded with CSS [34]. More specifically, preferred CSS are used, which provides best cross-correlation properties for this kind of sequences [35]. As the system consists of four buoys, four preferred CSS are needed. Every buoy emits a complementary set of four sequences, each of them of length 64. The sequences are interleaved and modulated in BPSK with a carrier frequency of 20 *kHz*, giving a total emission time of 12.8 *ms*.

**Figure 3.**
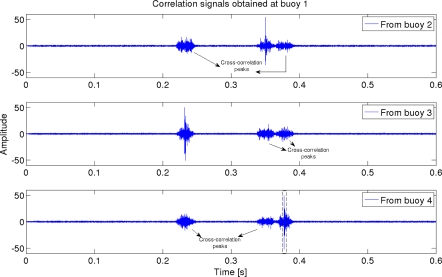
Correlation peaks at fixed buoy 1.

**Figure 4.**
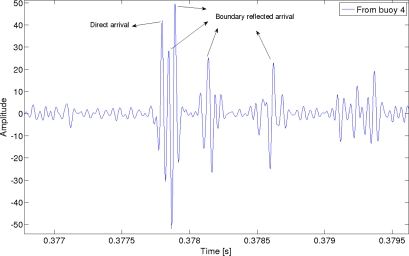
Autocorrelation peaks at buoy 1 with the code from buoy 4.

At each buoy, the received signal consists of the signals coming from the other three, which have suffered a fading obtained with the propagation model. Every buoy correlates this received signal with the CSS codes of the other three buoys, obtaining several correlation peaks, as can be seen in Figure 3, that shows an example for the signal received at buoy 1. Cross-correlation peaks have been marked in the figure, whereas Figure 4 shows a zoom of the autocorrelation peaks with the code from buoy 4. Several peaks can be detected, the first one coming from the direct arrival, and the other ones are boundary reflected paths. One of them has higher amplitude than the direct path, due to the particular phase interference. This is one of the sources of error in the measurement of the times of flight.

The measure of the time-of-flight (TOF) is done from the maximum amplitude peak from the correlation function. As the correlation peak is obtained when the entire coded signal has passed through the correlator, the TOF between two buoys (*i, j*) will be the difference between the time at which the maximum amplitude peak is detected, *t_ij_*, and the duration of the emitted signal, *t_code_*. Knowing these TOFs and the sound speed value computed at the hydrophone depth, *c*(*z* = 1 *m*), the distance between these buoys, *d_ij_*, can be calculated by Equation (10). This formula does not provide errors for isovelocity profiles, like the one considered in this work, as it will be seen in Section 4.

(10) $$d_{\mathrm{ij}}=\left(t_{\mathrm{ij}}-t_{\mathrm{code}}\right)c(z=1m)$$

### 3.2. MDS Positioning Algorithm

The buoys position are obtained with the MDS positioning algorithm [36]. To use the MDS technique, all the distances between the buoys are needed. This distances are computed by Equation (10) and collected into the matrix **D**, as seen in Equation (11),

(11) $$D=\left(\begin{array}{llll} d_{11} & d_{12} & d_{13} & d_{14}\\ d_{21} & d_{22} & d_{23} & d_{24}\\ d_{31} & d_{32} & d_{33} & d_{34}\\ d_{41} & d_{42} & d_{43} & d_{44} \end{array}\right)$$

where *d_ij_* = *d_ji_*, so the average of the distances from buoy *i* to *j* and *j* to *i* is used in matrix **D** for each symmetrical elements.

To obtain the buoys positions, it is necessary to build another matrix **H**, called dot-product, whose elements *H_ij_* are obtained by Equation (12):

(12) $$H_{\mathrm{ij}}=-\frac{1}{2}\left(d_{\mathrm{ij}}^{2}-\frac{1}{Q}\sum_{q=1}^{Q}d_{\mathrm{iq}}^{2}-\frac{1}{Q}\sum_{l=1}^{Q}d_{\mathrm{lj}}^{2}+\frac{1}{Q^{2}}\sum_{m=1}^{Q}\sum_{n=1}^{Q}d_{\mathrm{mn}}^{2}\right)$$

where *Q* is the number of buoys. By using Singular Value Decomposition (SVD), this matrix **H** can be related to the positions of the buoys referred to the centroid of the figure by Equation (13):

(13) $$H=\left(U\cdot S^{1/2}\right)\cdot {\left(U\cdot S^{1/2}\right)}^{T}$$

where **U** is the eigenvector matrix and **S** is the eigenvalue matrix, obtained by the SVD, and

(14) $$p_{c}=\left(U\cdot S^{1/2}\right)$$

where **p***_c_* is the position matrix of the buoys, referred to the centroid of the figure that form the buoys in 2D or 3D. To obtain the final positions referred to the origin of the coordinate system, is is necessary to perform a rotation and a translation.

## 4. Results Using the Relative Positioning System

In this section, some simulated results using the relative positioning system are presented. A preliminary version of these results were presented at the OCEANS 2011 Conference [24], and the SAAEI 2011 Conference [37]. In this work, a statistical study of the performance of the system has been performed for different values of wind speed *w*, and SNR. Additionally, another set of simulations shows the tracking of two free-moving buoys at the surface for different simulation conditions.

The positioning system is simulated to be deployed in the coast of Comodoro Rivadavia, Argentina. The latitude of this city is −45.8647°, and it has a bottom depth of 6 *m*, few kilometers out to sea. This bottom is supposed to be sandy, with a density of 1, 941 *kgm*^−3^ and a sound speed of 1, 749 *ms*^−1^. A value of 1, 024 *kgm*^−3^ has been considered for the water density, as well as a salinity of 34.1‰ and a pH of 7. All these values are between the most common that one can encounter in the medium [38].

The value for the water temperature has been obtained by means of the Levitus Atlas [39]. The annual mean for the approximate latitude of Comodoro Rivadavia has been used, with a value of 8.1 °*C*. As the bottom depth is at 6 *m*, the system is deployed in a very shallow water environment, and thus, the temperature is assumed to be constant in all the water column, as well as the salinity, obtaining then a constant sound speed profile with the Chen–Millero equation.

### 4.1. Statistical Study of the System Behavior

Due to the statistical nature of the dynamic transfer function, a statistical study has been conducted. For each value of wind speed and SNR, a hundred simulations have been performed and the average error for each buoy has been obtained.

The positioning system consist of four buoys. The statistical study has been made considering only one position for all the buoys. The first buoy is fixed at (0, 0) *m*, whereas the second fixed buoy is placed at (0, 500) *m*. The position for the third buoy is (300, 125) *m* and for the fourth one, (450, 300) *m*.

**Figure 5.**
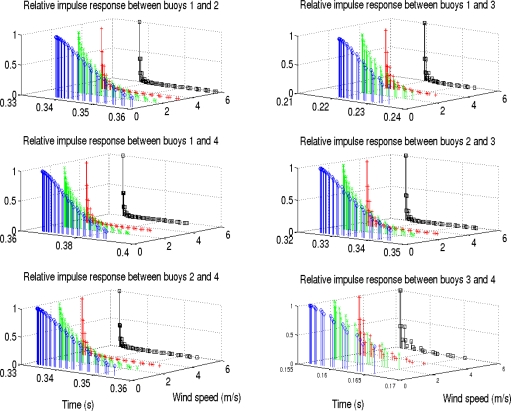
Dependence of the relative impulse response on wind speed.

In this work, the SNR is defined as *E_b_*/*N*_0_, where *E_b_* is the energy per bit and *N*_0_ is the noise power spectral density, assuming an additive white Gaussian noise. The SNR values used were 12, 0 and −6 *dB*, obtaining three different situations with a strong signal in the first case, a noticeable noise in the second one and a very unfavorable situation in the third one. For the wind speed, four values were used: 0.5, 2, 3.5 and 6 *ms*^−1^, considering different situations ranging from almost no wind to a remarkable wind speed between them, where these values are easily found in Comodoro Rivadavia. The effect of the wind in the impulse response and the time spread can be seen in Figure 5, where the responses defined by each pair of buoys have been represented. Interference is expected between the signals of different buoys, as a severe multipath is present when there is almost no wind speed.

**Figure 6.**
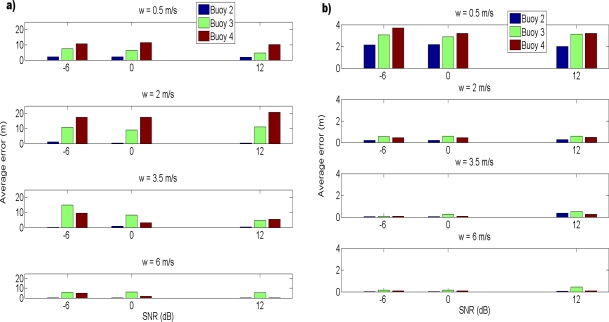
Average error of estimated positions for different values of SNR and wind speed: **(a)** with outliers, **(b)** without outliers. Note the different scales used in both Y axes.

The results for the average error in each buoy, considering different values of SNR and wind speed are shown in Figure 6, where the values for the first buoy are not given, as this buoy is the origin and it is always considered fixed at (0, 0) *m*. In order to avoid the late arrivals due to multipath, as well as the detection of a maximum from another signal due to the near-far effect, a threshold of 10 *m* has been considered. Errors greater than 10 *m* has been identified as outliers and removed, after checking the histograms to verify that most of the errors were below 10 *m*. Figure 6(a) shows the results for all the simulations, whereas in Figure 6(b) the outliers have been taken out. Note the different scales used in the Y axes of Figure 6(a,b).

The first conclusion that can be drawn from these results is that outliers mask the behavior of the system. These outliers are due to the near-far effect, when a cross-correlation peak has a greater amplitude than an autocorrelation peak, causing greater errors in value, of tens or hundreds of meters, as the peak detected is from another buoy. As can be seen in Figure 6(a), the largest errors are obtained for buoys 3 and 4. Those buoys are close, and so this effect is more noticeable in them.

More interesting conclusions can be drawn by removing the outliers. First of all, the errors are now lower, as can be seen in Figure 6(b). Additionally, SNR does not seem to be crucial in the performance of the system for the parameters studied, obtaining practically the same errors for the three values of SNR used. However, wind speed seems to be determinant. For greater values of wind speed, the average error tends to decrease. Note that the greater the wind speed, the more surface loss per rebound, and the less multipath signals in the received signal, making easier the detection of the correct peak.

### 4.2. Tracking of the Movement Due to a Surface Current

In the statistical study, all the buoys were considered to be in a constant position. In this case, the two free-moving buoys will vary their positions due to a surface current. The objective is to track the buoys and obtain their positions at each time. Knowing the distance traveled and the time difference between emissions, the velocity and direction of the surface current could be determined.

**Figure 7.**
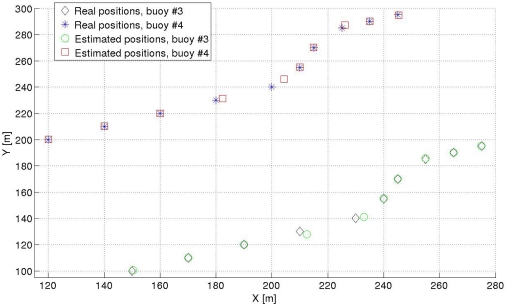
Buoys 3 and 4 movement due to a surface current.

**Figure 8.**
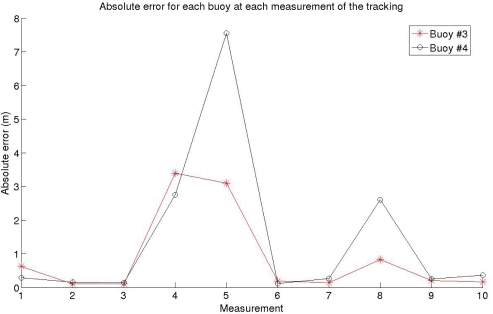
Absolute error for each buoy at each measurement.

Both the SNR and the wind speed can vary from one position to the next one, as well as the value and direction of the surface current, to represent a realistic as possible situation. The fixed buoys were placed at (0, 0) *m* and (0, 300) *m*, and the moving buoys were placed initially at (150, 100) *m* and (120, 200) *m*. The results of the tracking can be seen at Figure 7, whereas Figure 8 shows the absolute error for each position and each buoy. The values for the SNR, wind speed *w*, and surface current for each measure are given in Table 2.

As shown in Figure 7, the relative positioning system is capable to correctly obtain the position of the buoys. At some specific positions, measurements 4 and 5 of the tracking, the error is between 2 and 6 meters greater, as can be clearly seen in Figure 8. This error is due to the low wind speed considered at that particular moment, as can be read in Table 2, causing that the received signal is highly affected by multipath. In the position number 8 there is also a low value of wind speed, which also provides an inaccuracy of 2 *m* in the worst case in the measurement of the buoys position, as shown in Figures 7 and 8. From measurement number 2 to number 3, there is a noticeable fall on the SNR (9 *dB*), but the system keeps working properly, showing that the SNR is not an important parameter for the values considered. Additionally, it can also be seen that this system would be capable to detect changes in the direction of the surface current, as well as its numerical value.

## 5. Summary and Conclusions

An underwater acoustic propagation model has been proposed in this work. This model takes into account a sound speed profile, so it can be used both in shallow waters and deep waters. Additionally, the equations that are used to compute the sound speed and transmission loss are valid in a wide range of input values, so it is not restricted to very specific environmental conditions. It also considers the dynamic effect of swell that worsens the properties of the surface-reflected signal and uses a dynamic transfer function.

Also, a relative positioning system has been presented. The performance of this system has been studied using the propagation model described before. For a particular position, the average error has been obtained for each buoy varying the SNR and the wind speed. Multipath caused by low wind speeds has been identified as the most damaging effect, and some outliers have been detected, due to the near-far effect, which disguise the behavior of the system.

As an early example of application, the relative positioning system has been used to track the position of the buoys, where two of them can freely move through the sea surface due to surface currents. The system is capable to track the positions in different times, so the value and direction of the surface current could be computed. Using the MDS algorithm, the error in these measurements is about 1 *m* usually, and 8 *m* in the worst case, unless outliers appear. No outliers appeared for the tracking of the moving buoys.

As future work, some improvements can be made into the model, like adding a bathymetry profile or a range-dependent option, so the performance of this system could be studied in more complex environments. The tracking of the surface buoys must be estimated continuously, so a fast algorithm with low computational complexity has been used (MDS). The outliers could be reduced by implementing refinement algorithms, which will increase the complexity of the system to the detriment of the time between measurements. Finally, it is important to remark that although the tracking of moving buoys in the surface is interesting, this is not the final application of this system. That would be the simultaneous positioning of buoys in the surface and submerged objects in the sea, where acoustic systems can work in more complex environments than other wireless techniques, whose use is severely limited underwater.

## Figures and Tables

**Table 1.** Parameters for calculating the Chen–Millero equation [26].

Parameter	Value	Parameter	Value	Parameter	Value

*C*_00_	1402.388	*C*_01_	5.03830	*C*_02_	−5.81090E-2
*C*_03_	3.3432E-4	*C*_04_	−1.47797E-6	*C*_05_	3.1419E-9
*C*_10_	0.153563	*C*_11_	6.8999E-4	*C*_12_	−8.1829E-6
*C*_13_	1.3632E-7	*C*_14_	−6.1260E-10	*C*_20_	3.1260E-5
*C*_21_	−1.7111E-6	*C*_22_	2.5986E-8	*C*_23_	−2.5353E-10
*C*_24_	1.0415E-12	*C*_30_	−9.7729E-9	*C*_31_	3.8513E-10
*C*_32_	−2.3654E-12	*A*_00_	1.389	*A*_01_	−1.262E-2
*A*_02_	7.166E-5	*A*_03_	2.008E-6	*A*_04_	−3.21E-8
*A*_10_	9.4742E-5	*A*_11_	−1.2583E-5	*A*_12_	−6.4928E-8
*A*_13_	1.0515E-8	*A*_14_	−2.0142E-10	*A*_20_	−3.9064E-7
*A*_21_	9.1061E-9	*A*_22_	−1.6009E-10	*A*_23_	7.994E-12
*A*_30_	1.100E-10	*A*_31_	6.651E-12	*A*_32_	−3.391E-13
*B*_00_	−1.922E-2	*B*_01_	−4.42E-5	*B*_10_	7.3637E-5
*B*_11_	1.7950E-7	*D*_00_	1.727E-3	*D*_10_	−7.9836E-6

**Table 2.** Parameters used in the simulation.

Measurement	SNR (dB)	w (*ms*^−1^)	Surface current (*ms*^−1^)

1	6	2.5	(0.11,0.06)
2	6	2.5	(0.11,0.06)
3	−3	2.5	(0.11,0.06)
4	6	0.6	(0.11,0.06)
5	−6	0.6	(0.06,0.08)
6	3	4	(0.03,0.08)
7	3	4	(0.06,0.08)
8	3	1	(0.06,0.03)
9	6	2	(0.06,0.03)
10	6	2	(0,0)
